# The Synthesis, Characterization, Molecular Docking and In Vitro Antitumor Activity of Benzothiazole Aniline (BTA) Conjugated Metal-Salen Complexes as Non-Platinum Chemotherapeutic Agents

**DOI:** 10.3390/ph15060751

**Published:** 2022-06-15

**Authors:** Md. Kamrul Islam, Seongmin Ha, Ah-Rum Baek, Byeong-Woo Yang, Yeoun-Hee Kim, Hyun-Jin Park, Minsup Kim, Sung-Wook Nam, Gang-Ho Lee, Yongmin Chang

**Affiliations:** 1 Institute of Biomedical Engineering Research, Kyungpook National University, 680, Gukchaebosang-ro, Jung-gu, Daegu 41944, Korea; mkislam2008@yahoo.com (M.K.I.); zx996574@gmail.com (S.H.); baxun@naver.com (A.-R.B.); byungwoo1128@naver.com (B.-W.Y.); 2 R&D Center, Etnova Therapeutics Corp., 124, Sagimakgol-ro, Jungwon-gu, Seongnam-si 13207, Korea; bigeye38@naver.com (Y.-H.K.); phj0808@etnova.co.kr (H.-J.P.); 3 InCerebro Drug Discovery Institute, Seoul 01811, Korea; minsupkim.bio@gmail.com; 4 Department of Molecular Medicine, School of Medicine, Kyungpook National University, 680, Gukchaebosang-ro, Jung-gu, Daegu 41944, Korea; nams@knu.ac.kr; 5 Department of Chemistry, Kyungpook National University, 80, Daehak-ro, Buk-gu, Daegu 41566, Korea; ghlee@knu.ac.kr; 6 Department of Medical & Biological Engineering, Kyungpook National University, 80, Daehak-ro, Buk-gu, Daegu 41566, Korea; 7 Department of Radiology, Kyungpook National University Hospital, 130 Dongdeok-ro, Jung-gu, Daegu 41944, Korea

**Keywords:** benzothiazole aniline, transition metals, salen ligands, DNA, anticancer, Schiff-based

## Abstract

Here, we describe the synthesis, characterization, and in vitro biological evaluation of a series of transition metal complexes containing benzothiazole aniline (BTA). We employed BTA, which is known for its selective anticancer activity, and a salen-type Schiff-based ligand to coordinate several transition metals to achieve selective and synergistic cytotoxicity. The compounds obtained were characterized by NMR spectroscopy, mass spectrometry, Fourier transform infrared spectroscopy, and elemental analysis. The compounds **L**, **MnL**, **FeL**, **CoL**, and **ZnL** showed promising in vitro cytotoxicity against cancer cells, and they had a lower IC_50_ than that of the clinically used cisplatin. In particular, **MnL** had synergistic cytotoxicity against liver, breast, and colon cancer cells. Moreover, **MnL**, **CoL**, and **CuL** promoted the production of reactive oxygen species in HepG2 tumor cell lines. The lead compound of this series, **MnL**, remained stable in physiological settings, and docking results showed that it interacted rationally with the minor groove of DNA. Therefore, **MnL** may serve as a viable alternative to platinum-based chemotherapy.

## 1. Introduction

Cisplatin [cis-diamminedichloroplatinum (II)] has played a central role in cancer treatment due to its established actions against a variety of cancers (such as testicular, ovarian, bladder, head and neck, esophageal, small and non-small cell lung, breast, cervical, stomach, and prostate cancers), resulting in increased research into transition metal complexes for use in cancer therapeutics [1,2]. Cisplatin causes DNA damage, repair, and cell death in various cell types by forming covalent bonds with purine base pairs in DNA [3,4]. However, adverse effects such as alopecia, ototoxicity, gastrointestinal toxicity, and haematological toxicity limit its clinical utility [5,6,7]. Moreover, non-selective modes of action and drug-resistance phenomena lower the impact of the agents [8,9,10]. Platinum (II) complexes may interfere with the receptor, enzyme, and protein activities, causing functional protein damage, and they may result in neurotoxicity [11,12]. These unresolved issues have led researchers to design non-platinum metal chelates as safe and effective anticancer therapies, although no non-platinum metal anticancer drug has completed clinical trials [13,14].

Metal ions and ligand types affect the efficacy of metal-based anticancer treatments [15]. Transition metals, which belong to the “d” block of the periodic table and found in groups III–XII, have a notable impact on the development of metal-based anticancer drugs [16,17]. Moreover, transition metals have a variety of oxidation states, and they may interact with various negatively charged molecules, thus aiding the discovery of metal-based medications with promising pharmacological efficacy [18,19]. Several reviews have described the properties of a few non-platinum metal complexes and their use in current cancer treatment [20,21]. Biologically active transition metal Schiff-based complexes have gained considerable interest because of their unique structural and electronic features and potent antiproliferative properties [22,23]. In particular, salen [N, N-bis(salicylidene)-1,2-ethylenediamine] type ligands have emerged as a drug target in the fight against cancer [24,25] because salen-type Schiff-based ligands comprising donor atoms, such as N and O, have a broad spectrum of biological functions and are of particular interest because they are bonded with metal ions [26,27]. For example, Lee et al. showed that Schiff-based iron complexes could overcome MDR (multidrug resistance) in vincristine and daunorubicin-resistant Nalm-6 cells in vitro and ex vivo [28]. Similarly, Gust et al. developed cobalt (3,4-diarylsalen) chelates and tested their antitumor activity against the MDA-MB 231, MCF-7, and LNCaP/FGC cell lines [29]. Other studies have reported the anticancer properties of nickel (II) salophene (methoxy substituted) derivatives, and [Ni-III (OMe-salophene)] was found to overcome vincristine drug resistance in BJAB and Nalm-6 cells [30]. Further, a Schiff-based ligand coordinated to a copper (II) complex was evaluated and shown to increase apoptosis and reduce tumor growth by suppressing NF-kB, reactive oxygen species (ROS) production, and autophagy [31]. Additionally, Guo and his colleagues synthesized Mn-salen chelates that enhanced cell death in human prostate cancer cells via AMPK activation and increased cell autophagy [32].

Attaching benzothiazole aniline (BTA) to a metal coordination cage is a viable option for obtaining both selective and cytotoxic complexes. BTA derivatives exhibited selective cytotoxic activity against various tumor cell lines with no signs of hormone dependence [33,34]. Several studies have been conducted to develop new BTA derivatives for use against cancer [35,36,37]. For instance, the ring-substituted BTA derivative 2 (4-amino-3-methylphenyl) benzothiazole (DF 203) [phortress NSC 710305] was established as an anticancer agent and tested as a prodrug in phase 1 clinical trials [33,35]. Stevens and his colleagues have developed a series of benzothiazole derivatives based on the core molecule 2-(4-aminophenyl) benzothiazole (BTA) [38,39,40]. A single molecule of the Gd-DO3A-BTA combination was also synthesized and tested as a theranostic agent against numerous cancer cell lines in vitro and in vivo [41]. Mavroidi et al. recently identified Pt (II) and Pd (II) chelates of BTA derivatives for cancer cell targeting; further, the compounds were less cytotoxic than the clinically used cisplatin [42]. More recently, we synthesized a few BTA derivatives and their Pt (II) complexes and investigated their cytotoxicity against various cancer and normal cell types, but no synergistic cytotoxicity was identified [43].

In this study, we designed and synthesized innovative metal-based drug candidates by exploiting the unique properties of the transition metal core to identify those that interacted with their molecular targets. We hypothesized that the conjugation of BTA to a salen-type Schiff-based ligand might result in compounds with selective and synergistic cytotoxic effects. In vitro screening was performed using various malignant and healthy cell lines, and stability was assessed in buffered aqueous solutions and cell culture conditions. Furthermore, intracellular ROS production in HepG2 cells was analyzed; and, in silico ligand-docking simulations were performed to evaluate the binding mechanism of the most active compounds to DNA.

## 2. Results and Discussion

### 2.1. Synthesis and Characterization

**Scheme 1:**
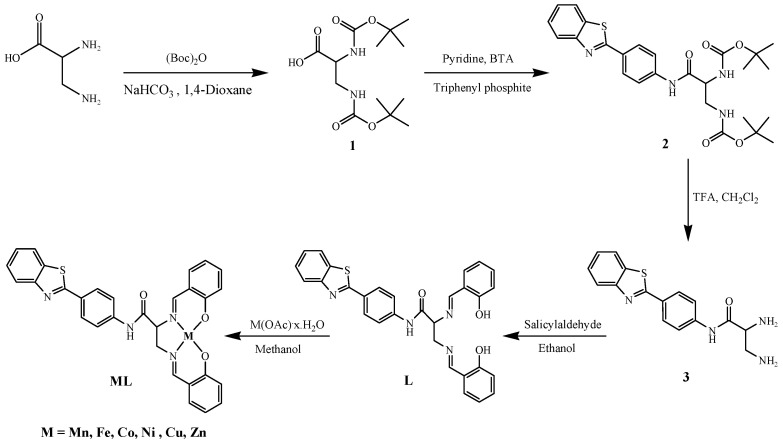
Synthesis of transition metal complexes.

The synthesis procedures for the ligand (**L**) and metal complexes (**ML**) are depicted in Scheme 1. All compounds were highly soluble in DMSO and stable in the presence of oxygen and moisture, enabling long-term storage without degradation.

**Figure 1:**
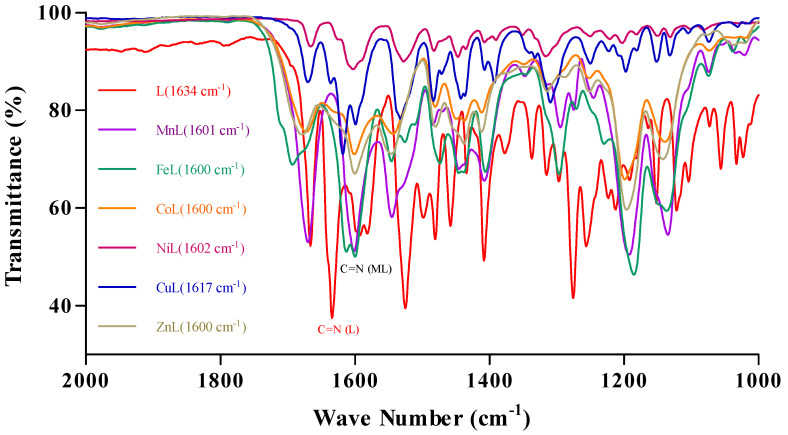
The FTIR spectrum of the Schiff’s base ligand (**L**) and transition metal complexes. The FTIR spectra indicated the presence of a C=N stretching vibration at 1634 cm^−1^ for the ligand (**L**) and a C=N stretching vibration downshift to 1600–1617 cm^−1^, showing the coordination of Schiff bases and metal ions.

All compounds were identified using NMR, Fourier transform infrared (FTIR), elemental analysis, and mass spectroscopy. The FTIR spectrum confirmed ligand (**L**) synthesis by removing the NH_2_ vibration and identifying a C=N stretching vibration at 1634 cm^−1^ (Figure 1 and Figure S2). The absence of the salicylic effect caused a downshift in the C=N stretching vibration to 1600–1617 cm^−1^, indicating that the Schiff bases and metal ions were coordinated (Figure 1 and Figures S4, S6, S8, S11, S13 and S16). The stretching vibration of the OH group at 3299 cm^−1^ was absent, indicating that phenolic oxygen was coordinated with the appropriate metal ions. The diamagnetic metal complexes **NiL** and **ZnL** were also investigated using ^1^H NMR spectroscopy; however, no ^1^H NMR spectra of **MnL**, **FeL**, **CoL**, or **CuL** were produced because of their substantial paramagnetism. The resonance signal of the OH protons of the ligand (**L**) was absent at frequencies of 12.09 and 12.8 ppm, and the aromatic proton’s resonance was shifted in different directions (Figures S1, S10 and S15). All compounds were analyzed using positive-ion MS, and parent peaks for **MnL**, **FeL**, **CoL**, **NiL**, **CuL**, and **ZnL** were observed at (*m*/*z*) 573, (*m*/*z*) 574, (*m*/*z*) 577, (*m*/*z*) 577, (*m*/*z*) 581, and (*m*/*z*) 622, respectively (Figures S5, S7, S9, S12, S14 and S17).

### 2.2. Complex Stability

**Figure 2:**
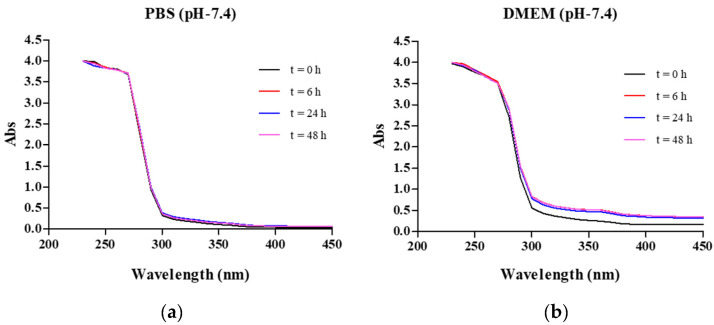
Time-dependent UV−Vis spectrum of the **MnL** complex in (**a**) PBS, and (**b**) DMEM cell culture medium at pH 7.4 and 25 °C in the 200−450 nm range.

Potential anticancer drugs should be stable in conditions similar to those found in living organisms. Therefore, we determined the relative stability of the complex in physiologically relevant conditions before conducting biological tests. The stability of the most active **MnL** complex was determined using UV-Vis spectroscopy. In DMSO, all complexes were soluble and stable. The stability of the **MnL** complex was evaluated over 48 h at room temperature by monitoring its UV-visible spectrum at 200–450 nm. As shown in Figure 2a, no substantial change in the spectra was observed, indicating **MnL** was highly stable in buffered aqueous solutions.

In addition, the stability of **MnL** in biological media was evaluated using Dulbecco’s modified Eagle’s medium (DMEM) as a culture medium. Figure 2b shows that **MnL** maintained its characteristic absorption peaks over 48 h, indicating that the parent complex preserved its integrity. Our results are consistent with that of an earlier study, demonstrating that the complex is stable in the culture medium [44,45].

### 2.3. Antiproliferative Activity

**Figure 3:**
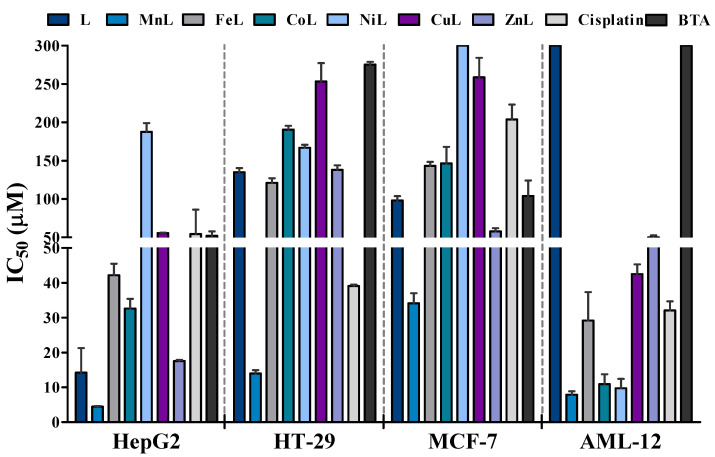
Graphical representation of IC_50_ values for **L**, transition metal complexes, cisplatin, and BTA in HepG2, HT-29, MCF-7, and AML-12 cell lines. The data are representative of the mean of three independent experiments (*n* = 3, mean ± SEM).

All compounds were tested by administering them to three cancer and one healthy cell line, HepG2 (hepatocellular carcinoma), HT-29 (human colon cancer), MCF-7 (human breast carcinoma), and AML-12 (non-malignant mouse liver cell line), using the CCK-8 assay. The survivability of the cell lines was investigated using variable ligand (**L**) amounts and its transition metal complexes **MnL**, **FeL**, **CoL**, **NiL**, **CuL**, and **ZnL** (Table 1 and Figure 3). The half-maximal inhibitory concentration (IC_50_) was calculated. Cisplatin and BTA were used as the control groups. Except for **NiL**, the compounds **L**, **MnL**, **FeL**, **CoL**, and **ZnL** showed higher cytotoxic activity than cisplatin and BTA in liver cancer cells, whereas **CuL** had similar anticancer activity to cisplatin and BTA in the same cells. In addition, **ZnL** showed a higher anticancer effect in liver and breast cancer cells than cisplatin and BTA, but it had a synergistic effect in breast cancer cells. **NiL** showed a considerable anticancer effect in colon cancer cells relative to BTA, though the effect was not as strong as that of cisplatin. The structure-activity reveals that the cytotoxicity properties of the **L** are greatly improved when the BTA moiety is conjugated with an electron-donating, hydroxyl group-containing salen ligand. Nevertheless, metal complexes in vitro anticancer efficacy varies based on the coordinated metal ions and cancer cell types. Among the complexes analyzed, **MnL** showed considerably more potent anticancer activity with synergistic cytotoxicity against cancer cell lines than cisplatin and its parent BTA. Furthermore, the IC_50_ value of **MnL** in liver cancer cells (HepG2) was 2.5, 3, 5.5, 7, 9.5, and 33.5 times greater than that of **L**, **ZnL**, **CoL**, **FeL**, **CuL**, and **NiL**, respectively. The compounds had the following antiproliferative action in vitro in HepG2 liver cancer cells: **MnL** > **L** > **ZnL** > **CoL** > **FeL**> BTA > cisplatin > **CuL** > **NiL**. This finding implies that our newly developed compounds, in particular **MnL**, may serve as a feasible alternative to current platinum-based chemotherapy agents to mitigate adverse effects. Further studies concerning the targets of the complexes (such as DNA interaction studies) and intracellular accumulation (drug uptake/efflux studies) may help to elucidate the effect of substituents on the cytotoxic effects.

Next, we estimated the compound selectivity index (SI), which is generally derived using a simple ratio of the IC_50_ values for healthy and malignant cells, with values greater than one indicating preferential selectivity for cancer cells [46,47]. In this study, compound selectivity was determined in HepG2 liver cancer cells using a non-malignant mouse liver cell line (AML-12) as a healthy cell model (Table 1). Interestingly, no cytotoxicity was observed in normal mouse liver hepatocyte cells at doses up to 300 µM for the ligand (**L**) or its precursor, BTA. Furthermore, the compounds **MnL**, **FeL**, and **ZnL** were less toxic to normal cells than liver cancer cell lines, indicating that they may be utilized to target cancer cells rather than normal cells. In contrast, clinically used cisplatin is more cytotoxic to normal liver cells than to liver tumor cells.

### 2.4. Intracellular ROS Generation

**Figure 4:**
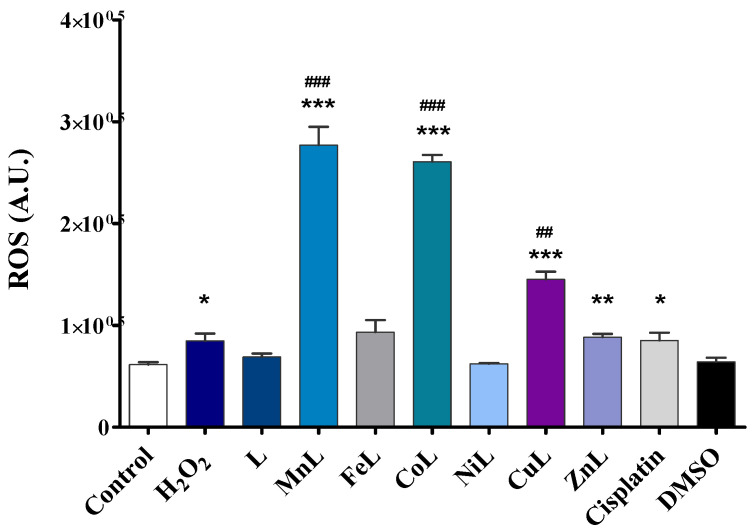
To measure ROS generation, the fluorescent probe H_2_DCF-DA was used. HepG_2_ cells were treated for 24 h with 250 µM H_2_O_2_, 50 µM metal complexes, and associated ligands. The data are presented as mean ± SEM (*n* = 3). * *p* < 0.05, ** *p* < 0.01, *** *p* < 0.001 relative to the control. ^##^ *p* < 0.01, ^###^ *p* < 0.001 relative to the H_2_O_2_.

Reactive oxygen species (ROS) are produced by cellular metabolism and play a critical role in maintaining cellular redox equilibrium. Increased levels of ROS cause damage to many cellular components, such as DNA, lipids, and proteins, and affect cellular function and induce apoptosis [48,49]. Anticancer drugs that stimulate ROS generation or block the antioxidant system are viable methods to kill cancer cells [50,51]. Therefore, we investigated the ability of the complexes to generate ROS using the H_2_DCF-DA assay to understand the mechanism underlying its inhibitory effect on cellular survival. The DCF-DA assay results showed that **MnL**, **CoL**, **CuL**, **ZnL**, and **FeL** generated more ROS than H_2_O_2_ and cisplatin in HepG2 cells, whereas **L** and **NiL** showed lower ROS generation than H_2_O_2_ and cisplatin (Figure 4). The amount of ROS production in HepG2 cells was in the following order: **MnL** > **CoL** > **CuL** > **ZnL** > **FeL** > cisplatin > **L** > **NiL**.

**Figure 5:**
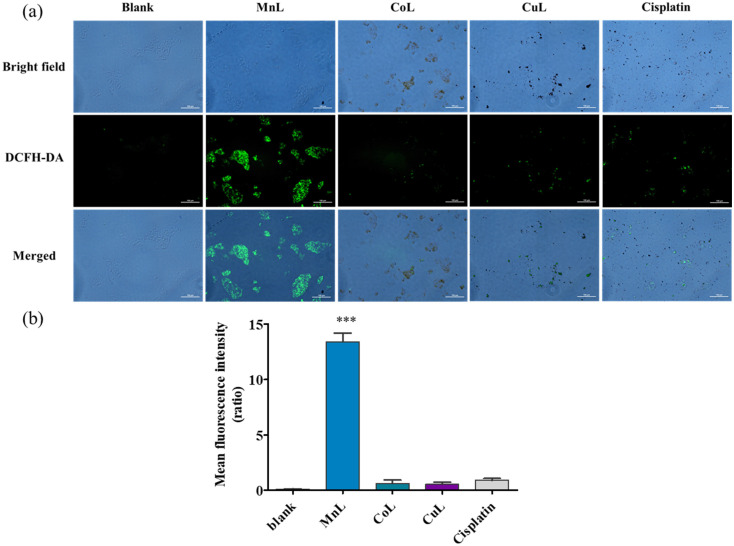
DCFH-DA staining to detect ROS in HepG2 cells treated with metal complexes. HepG2 cells were stained with DCFH-DA after 24 h of incubation with and without the metal complexes (blank: untreated group; metal complexes **MnL**, **CoL**, **CuL**, and cisplatin: IC_50_ values). (**a**) Representative micrographs for metal complexes and control. (**b**) Quantified ROS production data. The results are reported as the mean ± SEM (*n* = 3). *** *p* < 0.001 relatives to the blank.

HepG2 cells were treated with half-maximal inhibitory doses (IC_50_) of the compounds to analyze the change in intracellular ROS accumulation with the same number of live cells, and the mean fluorescence intensities of the active compounds were examined to calculate the overall ROS index (Figure 5). These data suggest that the **MnL**-treated group’s intracellular ROS levels increased more than those in the untreated group, other metal complexes, and commercially available cisplatin. Therefore, the results suggest that **MnL** causes HepG2 cells to be more susceptible to ROS-induced damage, resulting in cell death [52].

### 2.5. Ligand Docking Simulation of L, MnL, and BTA

Molecular docking is a useful computational tool for predicting drug–biomolecule interactions [53,54]. Ligand docking was performed to predict the binding mode of the most active compounds, **MnL**, **L**, and BTA, to DNA to assess the binding energy and the position of the compound inside DNA. The predicted binding energies of **L**, **MnL**, and BTA with diverse DNA topologies are presented in Table 2. As shown in Table 2, the compounds **L** and **MnL** showed binding energies of −7.239 and −5.894 kcal/mol to 1BNA, respectively, whereas the parent molecule BTA had a binding energy of −6.618 kcal/mol. The negative binding energy values indicated that DNA is a reasonable target for anticancer action. Similarly, **MnL** has a relative binding energy toward 1LU5 of −4.253 kcal/mol, which is similar to BTA (−4.259 kcal/mol). Moreover, in terms of docking binding energies, the compound **L** outperformed the corresponding metal complex **MnL** and parent BTA for the DNA topologies tested.

**Figure 6:**
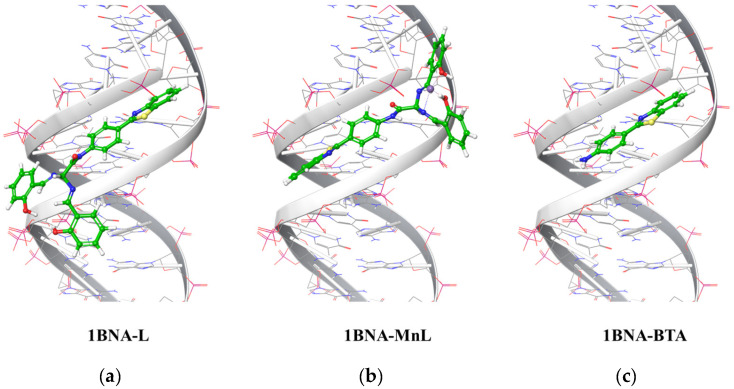
DNA binding configurations as predicted for the compounds (**a**) **L**, (**b**) **MnL**, and (**c**) BTA (PDB ID: 1BNA).

Intercalation, or major/minor groove binding with DNA, is the most prevalent interaction for small molecular anticancer agents [55]. The ligand in the groove binding mode is usually flexible, has rotatable bonds, and can position itself along the major or minor groove of the DNA, limiting normal DNA function. In contrast, DNA intercalators are often rigid planar molecules that stack between DNA base pairs, causing an intercalation gap in the helical structure of DNA. Based on the molecular modeling results, the most favorable docked pose of compounds **L** and **MnL** showed that they interact with the minor groove of DNA (PDB ID: IBNA) in a noncovalent manner (Figure 6). The BTA proportions of compounds **L** and **MnL** fit into the minor groove of DNA and interact hydrophobically with the base pairs. In contrast, the salen ligand is located away from the gap and interacts with DNA base pairs via π–π stacking through its –OH group. In addition, hydrogen bonds were established between the –NH group and DNA. Unlike their Mn complex counterparts, ligand **L** showed groove-binding intercalation with DNA, which explains the increased cytotoxicity observed. The interaction of the compounds **L**, **MnL**, and BTA with the 1LU5 and 3CO3 DNA structures are shown in the Supporting Information (Figures S18 and S19). However, we must emphasize that the docking study alone is insufficient to confirm the compound’s intercalating characteristics. An in vitro experiment, such as the ethidium bromide displacement assay, needs to be performed along with the docking study. Therefore, an additional study employing in vitro binding assays is required to investigate the mechanism of action of compounds.

## 3. Materials and Methods

### 3.1. Reagents and Instruments

All reagents and solvents were purified and dried using standard procedures. D, L-2, 3-Diaminopropionic acid monohydrobromide, di-tert-butyl dicarbonate, and triphenyl phosphite were obtained from Tokyo Chemical Industry (Tokyo, Japan). Sodium bicarbonate (NaHCO_3_) was supplied by Daejung Chemicals (Siheung-si, Korea). Magnesium sulfate (MgSO_4_) was obtained from Duksan Scientific Corp. (Ansan-si, Korea). Salicylaldehyde was obtained from Junsei Chemical Co., Ltd. (Korea). [2-(4-aminophenyl) benzothiazole] (BTA), and other commercial reagents were purchased from Sigma-Aldrich (St. Louis, MO, USA) unless stated otherwise. Compound **3** was synthesized using a previously described method [56,57], and it was subsequently reacted with two equivalents of salicylaldehyde to generate a white solid salen-type Schiff-based ligand (**L**). Additional treatment with appropriate metal salts resulted in the formation of analytically pure chelates as solid products. All experiments were performed using deionized water. TLC was used to monitor the reactions on 60 F254 silica gel plates (Merck, Darmstadt, Germany), and UV light was used to observe the reaction progress. ^1^H NMR experiments were performed using a Bruker Advance 500 spectrometer at the Center for Instrumental Analysis, Kyungpook National University (KNU), Daegu, South Korea. Chemical shifts were expressed in tetramethylsilane (TMS) as the internal standard, and coupling constants were defined in hertz (Hz). FTIR spectra were acquired in the 200–4000 cm^−1^ range using a KBr pellet on a Perkin–Elmer 883 twin-beam infrared spectrophotometer. The researchers at the Center for Instrumental Analysis at KNU in Daegu, Korea performed the elemental analyses. High-resolution fast atom bombardment mass spectra (HR-FAB-MS) were acquired at the Korea Basic Science Institute (KBSI) using a JMS-700 model mass spectrophotometer (Jeol, Japan). The Stanford OptiMelt MPA100, an automated melting point system, was used to determine the compounds’ melting points. Elemental analysis was performed to determine the purities of the compounds.

### 3.2. Synthesis and Characterization of Ligand

#### N-(4-Benzothiazol-2-yl-phenyl)-2,3-bis-[(2-hydroxy-benzylidene)-amino]-propionamide (L)

Compound (**L**) was prepared using a method described in a previous publication [43]. Briefly, salicylaldehyde (0.42 g, 3.5 mmol) was added dropwise to compound **3** (0.54 g, 1.7 mmol) and dissolved in ethanol (20 mL). The resulting mixture was refluxed for ~3 h until the starting ingredients were no longer visible. The resulting solid was filtered and washed with a small amount of cold ethanol, and the pure product was harvested as a white solid after resuspension in hexane. Yield: 48%. mp 150–152 °C; ^1^H NMR (CDCl_3_): δ = 12.8 (*s*, 2H, Ar-OH), 12.01 (*s*, 1H, NH), 8.45 (*s*, 1H, CH) 8.36 (*s*, 1H, CH), 8.07–8.01 (*m*, 3H, BTA), 7.90−7.86 (*d*, 1H, BTA), 7.71−7.66 (*d*, 2H, BTA), 7.49−7.44 (*t*, 1H, BTA), 7.41−7.38 (*t*, 1H, BTA), 7.36–7.25 (*m*, 3H, phe), 7.22–7.18 (*d*, 1H, phe) 7.04–6.81 (*m*, 4H, phe), 4.42−4.36 (*m*, 2H, CH_2_), 4.09–3.03 (*m*, 1H, CH). FTIR: v (cm^−1^) = 3299.9 w, 1665 s, 1634 s (C=N), 1524 s, 1407 s, 749 s. HR-FAB-MS (*m/z*): Calcd, 521.1647 [M + H]^+^; found, 521.1645 [M + H]^+^. Anal calcd for C_30_H_24_N_4_O_3_S·⅟_2_ H_2_O: H, 4.76; C, 68.06; N, 10.58; S, 6.06; found: H, 4.64; C, 68.10; N, 10.44; S, 6.08.

### 3.3. Synthesis and Characterization of Metal Complexes

The general procedure for the syntheses of transition metal complexes is as follows [58]: Ligand (**L**) (0.5 mmol) was dissolved in dry methanol (15 mL) and stirred overnight at rt in the presence of the respective metal salt (0.5 mmol). After repeated washing with methanol and ether, pure compounds were obtained as solid.

#### 3.3.1. Synthesis of MnL

Compound **MnL** was obtained from **L** (200 mg, 0.38 mmol) and manganese (II) acetate tetrahydrate (113 mg, 0.46 mmol). Yield: 39%. mp 229–231 °C; FTIR: v (cm^−1^) = 3062 w, 1601 s (C=N), 1543 s, 1435 m, 1197 s, 968 m, 754 s. ESI-MS (*m/z*): Calcd, 573.07 [M]^+^; found, 573.04 [M]^+^. Anal calcd for (C_30_H_22_MnN_4_O_3_S.4H_2_O): C, 55.81; H, 4.68; N, 8.68; S, 4.97, found: C, 55.40; H, 3.40; N, 9.33; S, 6.41.

#### 3.3.2. Synthesis of FeL

Compound **FeL** was obtained from **L** (400 mg, 0.77 mmol) and Fe (II) acetate tetrahydrate (190 mg, 0.77 mmol). Yield: 64.3%. mp 218–220 °C; FTIR: v (cm^−1^) = 3300 w, 1599 s (C=N), 1545 s, 1425 m, 1473 s, 1155 s, 753 s. HR-FAB-MS (*m/z*): Calcd, 574.0762 [M]^+^; found: 574.0765 [M]^+^. Anal calcd for C_30_H_24_FeN_4_O_4_S.3C_6_H_9_O_6_^3-^: C, 56.18; H, 4.32; N, 7.28; S, 4.17; found: C, 56.61; H, 3.56; N, 7.23; S, 4.25.

#### 3.3.3. Synthesis of CoL

Compound **CoL** was obtained from **L** (500 mg, 0.96 mmol) and Co (II) acetate tetrahydrate (296 mg, 1.15 mmol). Yield: 53.5%. mp 334–336 °C; FTIR: v (cm^−1^) = 3240 w, 1672 s, 1600 s (C=N), 1543 s, 1449 m, 1198 s, 970 s, 755 s. HR-FAB-MS (*m/z*): Calcd, 577.0745 [M]^+^ and 578.0823 [M + H]^+^; found: 577.0749 [M]^+^ and 578.0820 [M + H]^+^. Anal calcd for C_30_H_22_CoN_4_O_3_S·1.5H_2_O: C 59.61, H 4.15, N 9.27, S 5.30; found: C 59.34, H 3.78, N 9.13, S 5.54.

#### 3.3.4. Synthesis of NiL

Compound **NiL** was obtained using **L** (300 mg, 0.58 mmol) and Ni (II) acetate tetrahydrate (216 mg, 0.87 mmol). Yield: 52.6%. mp 340–342 °C; ^1^H NMR (500 MHz, DMSO-d_6_): δ = 10.60 (*s*, 1H, NH), 8.14–8.06 (*dd*, 3H, BTA), 8.02 (*t*, 2H, CH), 7.90 (*s*, 1H, BTA), 7.86−7.83 (*d*, 2H, BTA), 7.53 (*t*, 1H, BTA), 7.44 (*t*, 1H, BTA), 7.29−7.15 (*m*, 4H, phe), 6.75−6.71 (*m*, 2H, phe), 6.56–6.48 (*m*, 2H, phe), 4.31 (*m*, 1H, CH), 4.10 (*m*, 1H, CH_2_), 3.98 (*m*, 1H, CH_2_). FTIR: v (cm^−1^) = 3010 w, 1665 s, 1602 s (C=N), 1527 s, 1446 m, 1316 m, 964 s, 749 m. Anal calcd for C_30_H_22_N_4_NiO_3_S: C 62.42, H 3.84, N 9.71, S 5.55; found: C 62.18, H 3.90, N 9.16, S 5.46. ESI-MS (*m/z*): Calcd, 577.08 [M + H]^+^; found, 577.05 [M + H]^+^.

#### 3.3.5. Synthesis of CuL

Compound **CuL** was obtained from **L** (700 mg, 1.35 mmol) and copper (II) acetate monohydrate (270 mg, 1.35 mmol). Yield: 79%. mp 306–307 °C; FTIR: v (cm^−1^) = 3054 w, 1669 s, 1617 s (C=N), 1532 s, 1442 m, 1309 m, 752 s. HR-FAB-MS (*m/z*): Calcd, 581.08 [M]^+^; found, 581.37 [M]^+^. Anal calcd for C_30_H_22_CuN_4_O_3_S·H_2_O: C, 60.04; H, 4.03; 9.34; S, 5.34 Found: C, 59.84; H, 3.74; N, 9.33; S, 5.51.

#### 3.3.6. Synthesis of ZnL

Compound **ZnL** was obtained from **L** (200 mg, 0.38 mmol) and zinc (II) acetate dihydrate (113 mg, 0.46 mmol). Yield: 39%. mp 202–203 °C; ^1^H NMR (500 MHz, DMSO-d_6_): δ = 10.41 (*s*, 1H, NH), 8.51–8.44 (*dd*, 1H, CH), 8.13–8.06 (*m*, 5H, CH), 7.83 (*s*, 2H, CH), 7.55−7.46 (*d*, 3H, CH), 6.98–6.90 (*m*, 2H, CH), 6.73–6.62 (*d*, 1H, CH), 6.47 (*m*, 2H, CH), 6.30 (*m*, 2H, CH), 4.61 (*m*, 1H, CH), 4.02 –3.92 (*m*, 2H, CH_2_). FTIR: v (cm^−1^) =3064 w, 1678 s, 1600 s (C=N), 1545 s, 1437 m, 1196 s, 970 m, 755 s. ESI-MS (*m/z*): Calcd, 622.04 [M + K]^+^; found, 622.99 [M + K]^+^. Anal calcd for C_34_H_28_N_4_O_7_SZn·5H_2_O: C, 51.55; H, 4.84; N, 7.07; S, 4.05; found: C, 51.52; H, 3.58; N, 6.98; S, 4.34.

### 3.4. Stability of the Lead Complex

The complex **MnL** (1 × 10^−2^ M) was first dissolved in a small amount of DMSO (20% of the final volume) before diluting with PBS. Next, a solution of complex **MnL** at a concentration of (1 × 10^−3^ M) in PBS and 62.5 μg mL^−1^ in the DMEM culture medium was prepared at 37 °C and subjected to time-dependent UV−Vis spectroscopic monitoring at 0, 6, 24, and 48 h after preparation. Time-dependent UV−Vis spectra in the 200–450 nm range were obtained.

### 3.5. Cell Culture

Human hepatocellular carcinoma cells (HepG2, ATCC^®^ HB-8065) and human breast adenocarcinoma cells (MCF-7, ATCC^®^ HTB-22™) were cultured in Eagle’s Minimum Essential Medium (EMEM; ATCC, Manassas, VA, USA) supplemented with 10% (*v*/*v*) fetal bovine serum (FBS; Gibco, Grand Island, NY, USA) and 1% antibiotic-antimycotic (Gibco). Human colorectal adenocarcinoma cells (HT-29, ATCC^®^ HTB-38) were cultured in the growth medium containing Roswell Park Memorial Institute Medium (RPMI1640, WelGENE, Daegu, Korea) supplemented with 10% (*v*/*v*) FBS and 1% antibiotic-antimycotic (Gibco). Mouse liver normal cells (AML-12, ATCC^®^ CRL-2254™) were cultured in DMEM/F-12 (WelGENE) supplemented with 10% FBS (Gibco), 1 × ITS (10 µg/mL insulin, 5.5 µg/mL transferrin, 6.7 ng/mL selenium, Gibco), 40 ng minimum essential medium Eagle (WelGENE) supplemented with 10% FBS (Gibco, Grand Island, NY, USA), and 1% antibiotic-antimycotic (Gibco). Cells were incubated in a humidified atmosphere containing 5% CO_2_ at 37 °C.

### 3.6. Cell Viability Assay

To evaluate cytotoxicity, cells were seeded in 96-well plates (HepG2, HT-29, and MCF-7 cells at 2 × 10^4^ cells/well; AML-12 cells at 1.2 × 10^4^ cells/well). After adhering and stabilizing the cells for 24 h, the medium was changed to contain different concentrations of cisplatin (Sigma-Aldrich), BTA (Sigma-Aldrich), **L**, **MnL**, **FeL**, **CoL**, **NiL**, **CuL**, or **ZnL**, and the cells were incubated for 22 h. Following the completion of the treatment, a cell counting kit-8 (CCK-8, Dojindo Laboratories, Kumamoto, Japan) solution was added to each well plate and incubated for an additional 2 h. A microplate reader (SpectraMax i3; Molecular Devices, San Jose, CA, USA) was used to measure the absorbance at 450 nm, and the IC_50_ and logIC_50_ values were computed using GraphPad Prism (GraphPad Prism Software Inc. Version 5.02, San Diego, CA, USA). All experiments were repeated thrice, and the graph of the logIC_50_ values represents the average value.

### 3.7. Intracellular ROS Analysis

The fluorescent probe 2′,7′-dichlorodihydrofluorescein diacetate (H_2_DCF-DA) was used to evaluate ROS levels. H_2_DCF-DA monitors total ROS (peroxyl and hydroxyl radicals) generation in live cells. After passing through the cell membrane, intracellular esterases deacetylate H_2_DCF-DA, resulting in a non-fluorescent (H_2_DCF) probe, which ROS promptly oxidizes into the highly fluorescent 2′,7′-dichlorofluorescein (DCF) [52]. The amount of DCF produced is proportional to the fluorescence signal generated by ROS production. Based on the cytotoxicity assay results, 50 µM of the ligand and transition metal complexes were used for treatment. A 96-well plate containing 1.2 × 10^4^ cells/well were incubated for 24 h at 37 °C with 5% CO_2_ before being treated for 1 h with H_2_DCF-DA (25 µM). The cells were subsequently rinsed with HBSS before being treated at 37 °C for 24 h with the 50 µM concentration of the compounds or 250 µM of H_2_O_2_ (positive control). The fluorescence intensity was measured using an EnSpire Multimode Plate Reader (PerkinElmer, Waltham, MA, USA) at an excitation wavelength of 498 nm and an emission wavelength of 522 nm to determine intracellular ROS levels.

The H_2_DCF-DA ROS probe was used, and cells were observed under a fluorescence microscope to measure intracellular ROS levels. The assay quantifies ROS in living cells using a 2′,7′-dichlorofluorescein diacetate (DCFDA) probe, and it can be used as a marker for drug-induced ROS generation. The cells (6.5 × 10^4^ cells/well) were plated on 4-well culture slides and incubated overnight. The next day, compounds with half-maximal inhibitory concentrations were added. After 24 h, the cells were rinsed with HBSS and incubated with a medium containing 10 μM H_2_DCF-DA for 20 min at 37 °C. After incubation, the cells were mounted with an antifade mounting medium (VECTASHIELD, Vector Laboratories, Inc., Burlingame, CA, USA). Images were obtained using a fluorescence microscope (ECLIPSE Ti, Nikon, Melville, NY, USA).

### 3.8. Computational Method for Predicting Ligand-DNA Complexes

The binding conformations and energies of **L**, **MnL**, and BTA for DNA structures were predicted using a protein-ligand docking simulation application called Glide [59]. Glide identifies potential binding conformations of the given ligands, and it calculates binding energies on the DNA helical structure [59]. For the docking simulations, the OPLS3 force field describes the atomic interactions between the ligand and DNA molecules. Flexible ligand sampling of the ligand was performed, and the standard precision mode was used.

### 3.9. Statistical Analysis

The GraphPad Prism software was used to perform statistical analyses (GraphPad Prism 5.03). The data were analyzed using a one-way analysis of variance followed by Tukey’s multiple comparison test. Data from cell-based assays are expressed as the mean ± standard error of the mean (SEM) from at least three independent experiments, and statistical significance was defined at *p* < 0.05.

## 4. Conclusions

We synthesized and evaluated a series of transition metal complexes containing benzothiazole aniline-conjugated Schiff-based ligands. All compounds were tested for in vitro cytotoxicity against cancer and normal cells using a CCK-8 assay. The lead compound of this series, **MnL**, synergistically inhibited liver, colon, and breast cancer cells compared to clinically used cisplatin and the parent compound BTA. In addition, **MnL** remained stable in physiologically relevant conditions and generated ROS in HepG2 cancer cells. Ligand docking simulations showed that **MnL** interacted with the minor groove of the DNA. Taken together, both DNA binding and ROS generation may influence the antiproliferative activity of transition metal complexes. Therefore, transition metal complexes containing benzothiazole aniline-conjugated Schiff-based ligands, especially **MnL**, may serve as promising alternatives to platinum-based chemotherapeutic agents.

## Figures and Tables

**Table 1:** Half inhibitory concentration (IC_50_) of the ligand and transition metal complexes in cancer and normal cells. For each cell line, the IC_50_ values are the averages of three independent experiments [(*n* = 3, mean ± SEM (standard error of the mean)]. Cisplatin and BTA were used as positive controls, and the selectivity index (SI) in HepG2 cells is shown.

Compounds	IC_50_ (μM)				Selectivity Index (SI) ^a^
	Human Liver Cancer (HepG2)	Human Colon Cancer (HT-29)	Human Breast Cancer (MCF-7)	Mouse Liver Hepatocyte (AML-12)	
**L**	14.2 ± 7.0	135.0 ± 4.4	98.1 ± 7.9	>300	21.13
**MnL**	5.8 ± 0.3	15.1 ± 1.1	34.5 ± 4.0	9.4 ± 0.8	1.62
**FeL**	41.2 ± 3.8	110.3 ± 17.6	142.0 ± 8.9	48.1 ± 0.7	1.17
**CoL**	32.1 ± 4.4	181.8 ± 16.4	146.5 ± 30.5	14.7 ± 0.5	0.46
**NiL**	193.6 ± 17.7	167.1 ± 5.8	>300	12.4 ± 0.6	0.06
**CuL**	55.5 ± 0.6	135.1 ± 10.0	271.2 ± 37.0	46.5 ± 0.8	0.84
**ZnL**	17.6 ± 0.4	254.8 ± 33.8	57.6 ± 5.8	52.6 ± 0.5	2.99
**Cisplatin ^b^**	54.2 ± 31.8	39.1 ± 0.6	203.8 ± 27.3	32.1 ± 4.4	0.59
**BTA ^b^**	52.0 ± 5.7	275.3 ± 5.0	103.9 ± 28.6	>300	5.77

^a^ SI = IC_50_ (AML12)/IC_50_ (HepG2). ^b^ Data used from reference [43].

**Table 2:** Predicted binding energies for **L**, **MnL**, and BTA with various DNA configurations.

Compounds	Docking Binding Energy (kcal/mol)		
	1BNA ^a^	1LU5 ^b^	3CO3 ^c^
**L**	−7.239	−6.307	−7.718
**MnL**	−5.894	−4.253	−3.340
**BTA**	−6.618	−4.259	−5.941

^a^ Structure of a B-DNA dodecamer. ^b^ Asymmetric platinum complex {Pt(ammine)(cyclohexylamine)}^2+^ bound to a dodecamer DNA duplex. ^c^ *cis*-{Pt(NH_3_)_2_(pyridine)}^2+^ bound to deoxyguanosine in a dodecamer duplex (a monofunctional platinum–DNA adduct).

## Data Availability

Data is contained within the article and Supplementary Materials.

## Supplementary Materials

The supporting materials are available online at https://www.mdpi.com/article/10.3390/ph15060751/s1, Figure S1: ^1^H NMR spectrum of compound **L**, Figure S2: FTIR spectrum of compound **L**, Figure S3: High resolution mass spectrum of compound **L**, Figure S4: FTIR spectrum of compound **MnL**, Figure S5: ESI mass spectrum of compound **MnL**, Figure S6: FTIR spectrum of compound **FeL**, Figure S7: High resolution mass spectrum of compound **FeL**, Figure S8: FTIR spectrum of compound **CoL**, Figure S9: High resolution mass spectrum of compound **CoL**, Figure S10: ^1^H NMR spectrum of compound **NiL**, Figure S11: FTIR spectrum of compound **NiL**, Figure S12: ESI mass spectrum of compound **NiL**, Figure S13: FTIR spectrum of compound **CuL**, Figure S14: High resolution mass spectrum of compound **CuL**, Figure S15: ^1^H NMR spectrum of compound **ZnL**, Figure S16: FTIR spectrum of compound **ZnL**, Figure S17: ESI mass spectrum of compound **ZnL**, Figure S18: Molecular docking of **L**, **MnL**, and BTA with DNA, Figure S19: Molecular docking of **L**, **MnL**, and BTA with DNA.
