# Supplementary material for: The Synthesis, Characterization, Molecular Docking and In Vitro Antitumor Activity of Benzothiazole Aniline (BTA) Conjugated Metal-Salen Complexes as Non-Platinum Chemotherapeutic Agents
Source: Pharmaceuticals (Basel). 2022 Jun 15;15(6):751. doi: 10.3390/ph15060751 (PMC9228978; doi:10.3390/ph15060751)
Supplement: Supplementary file 1 [file pharmaceuticals-15-00751-s001.zip › pharmaceuticals-1714658-supplementary.pdf]

Supporting Information

The Synthesis, Characterization, Molecular docking, and In Vitro Antitumour Activity of Benzothiazole Aniline (BTA) Conjugated Metal-Salen Complexes as Non-Platinum Chemotherapeutic Agents

Md. Kamrul Islam [1], Seongmin Ha [1], Ah-Rum Baek [1], Byeong-Woo Yang [1], Yeoun-Hee Kim [2], Hyun-Jin Park [2], Minsup Kim [3], Sung-Wook Nam [4], Gang-Ho Lee [5] and Yongmin Chang [4,6,7,*]

[1] Institute of Biomedical Engineering Research, Kyungpook National University, 680, Gukchaebosang-ro, Jung-gu, Daegu 41944, Republic of Korea

[2] R&D Center, Etnova Therapeutics Corp., 124, Sagimakgol-ro, Jungwon-gu, Gyeonggi-do 13207, Republic of Korea

[3] InCerebro Drug Discovery Institute, Seoul 01811, Republic of Korea

[4] Department of Medical & Biological Engineering, Kyungpook National University, 80, Daehak-ro, Buk-gu, Daegu 41566, Republic of Korea

[5] Department of Chemistry, Kyungpook National University, 80, Daehak-ro, Buk-gu, Daegu 41566, Republic of Korea

[6] Department of Molecular Medicine, School of Medicine, Kyungpook National University, 680, Gukchaebosang-ro, Jung-gu, Daegu 41944, Republic of Korea

[7] Department of Radiology, Kyungpook National University Hospital, 130 Dongdeok-ro, Jung-gu, Daegu 41944, Republic of Korea

* Correspondence: ychang@knu.ac.kr; Tel.: (+) 82-53-420-5471

Table of contents

1.	**Figure S1.** ^{1}H NMR spectrum of compound **L**.	S3
2.	**Figure S2.** FTIR spectrum of compound **L**.	S3
3.	**Figure S3.** High-resolution mass spectrum of compound **L**.	S4
4.	**Figure S4.** FTIR spectrum of compound **MnL**.	S4
5.	**Figure S5.** ESI mass spectrum of compound **MnL**.	S5
6.	**Figure S6.** FTIR spectrum of compound **FeL**.	S5
7.	**Figure S7.** High-resolution mass spectrum of compound **FeL**.	S6
8.	**Figure S8.** FTIR spectrum of compound **CoL**.	S6
9.	**Figure S9.** High-resolution mass spectrum of compound **CoL**.	S7
10.	**Figure S10.** ^{1}H NMR spectrum of compound **NiL**.	S7
11.	**Figure S11.** FTIR spectrum of compound **NiL**.	S8
12.	**Figure S12.** ESI mass spectrum of compound **NiL**.	S8
13.	**Figure S13.** FTIR spectrum of compound **CuL**.	S9
14.	**Figure S14.** High-resolution mass spectrum of compound **CuL**.	S9
15.	**Figure S15.** ^{1}H NMR spectrum of compound **ZnL**.	S10
16.	**Figure S16.** FTIR spectrum of compound **ZnL**.	S10
17.	**Figure S17.** ESI mass spectrum of compound **ZnL**.	S11
18.	**Figure S18.** Molecular docking of **L**, **MnL**, and BTA with DNA.	S11
19.	**Figure S19.** Molecular docking of **L**, **MnL**, and BTA with DNA.	S12

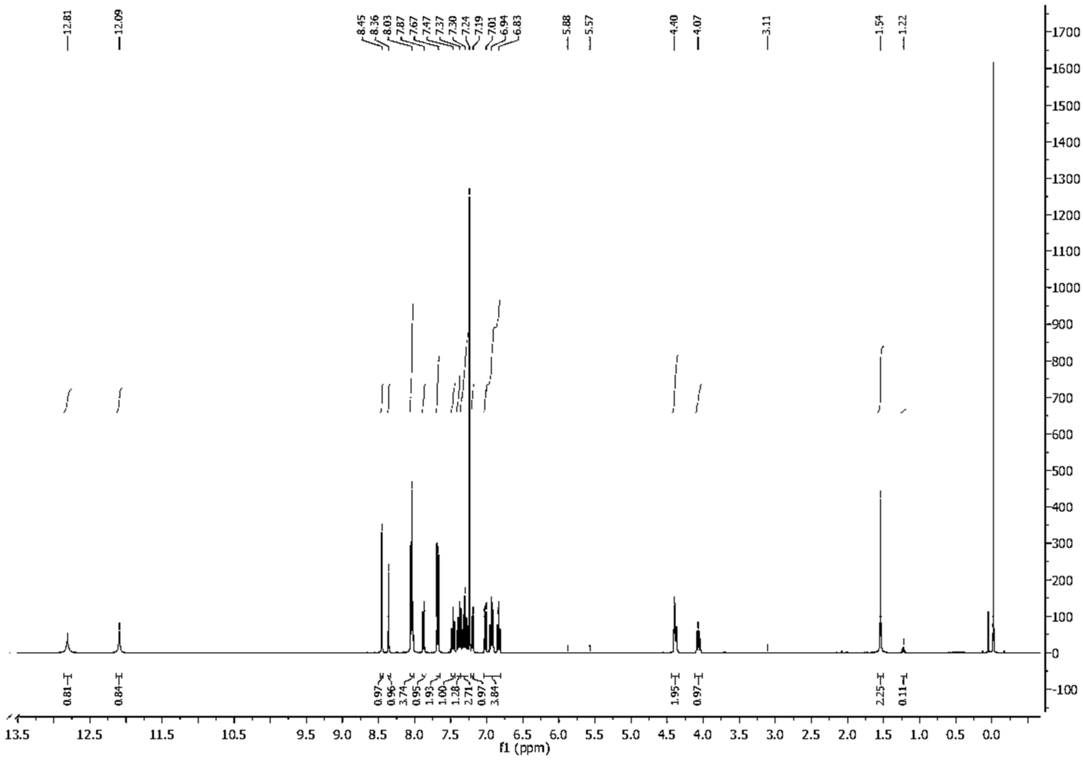

Figure S1. ^{1}H NMR spectrum of compound **L**.

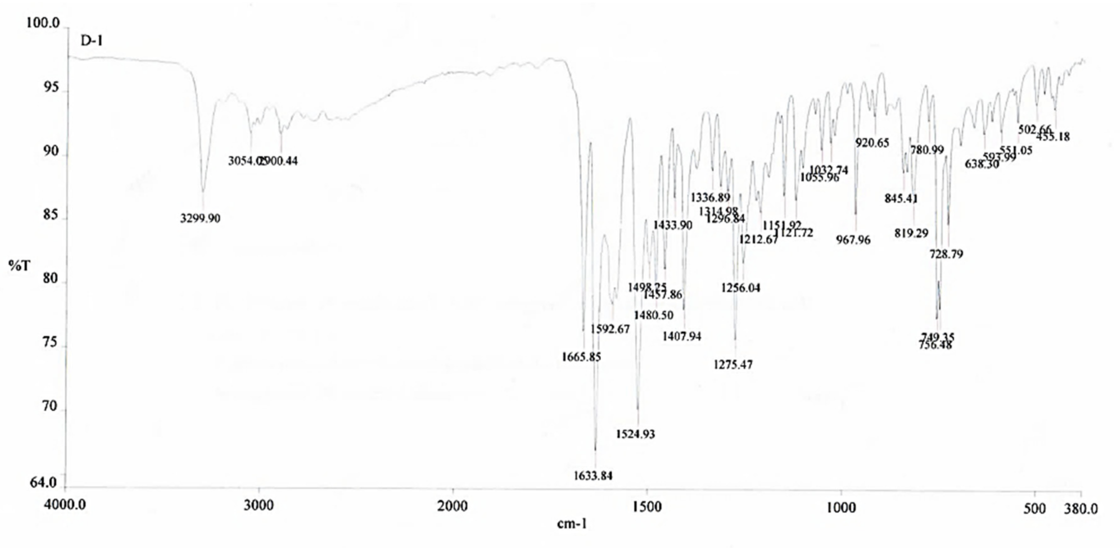

Figure S2. FT-IR spectrum of compound **L**.

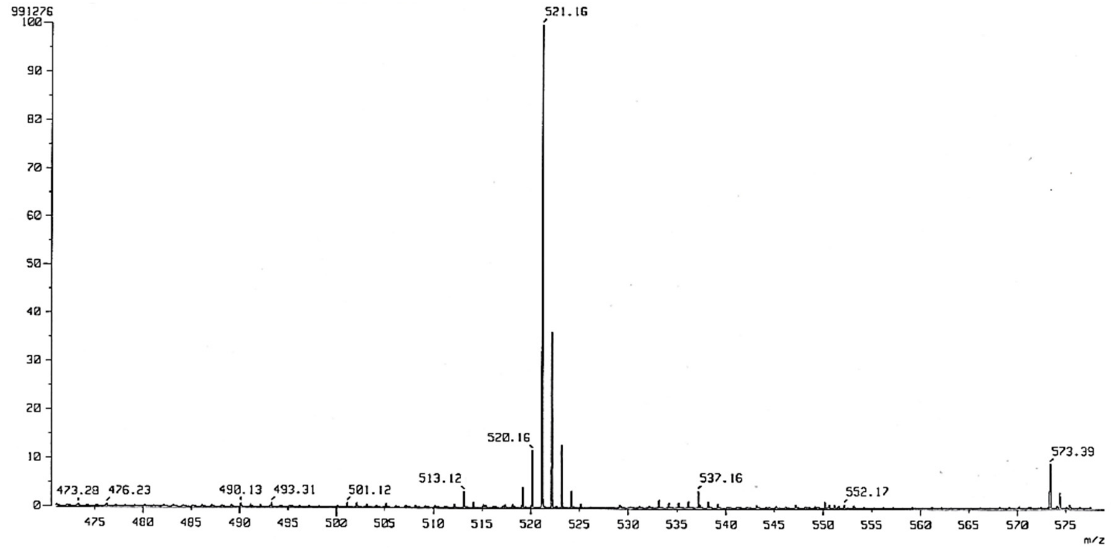

Figure S3. High-resolution FAB mass spectrum of compound **L**. Mass spectrum of main peak display parent ion (m/z): calcd, 521.1647 $[M+H]^+$; found, 521.1649 $[M+H]^+$.

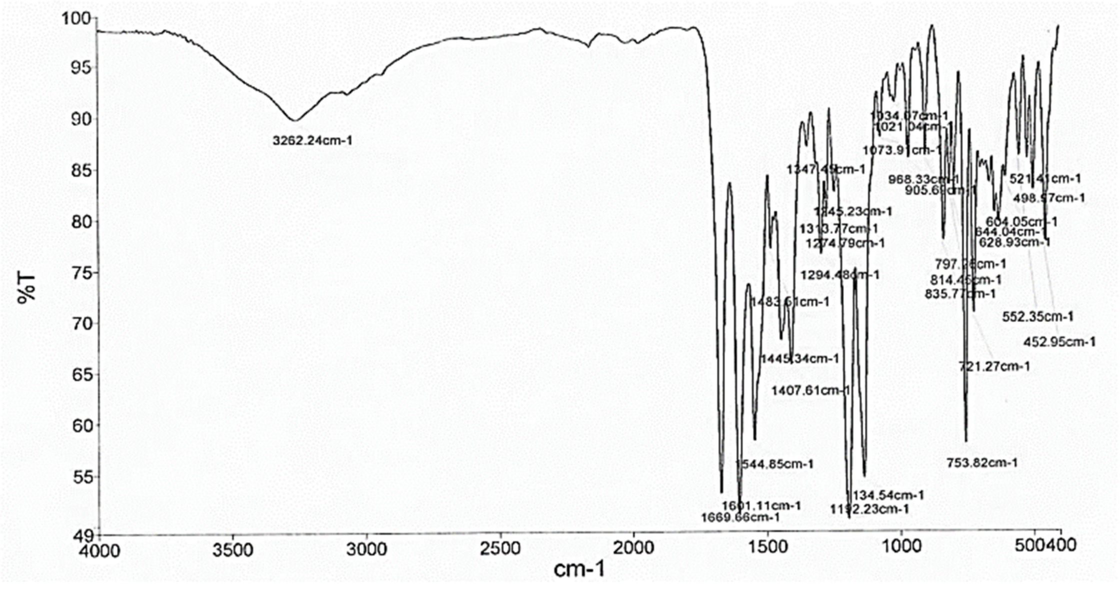

Figure S4. FTIR spectrum of compound **MnL**.

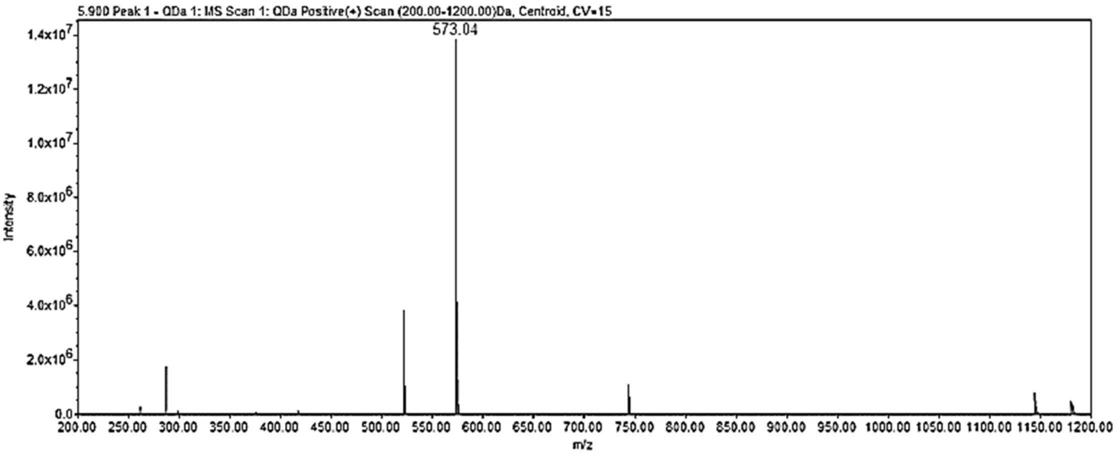

Figure S5. ESI-MS spectrum of compound **MnL**. Mass spectrum of main peak display parent ion (m/z): calcd, 573.07 $[M]^+$; found, 573.04 $[M]^+$.

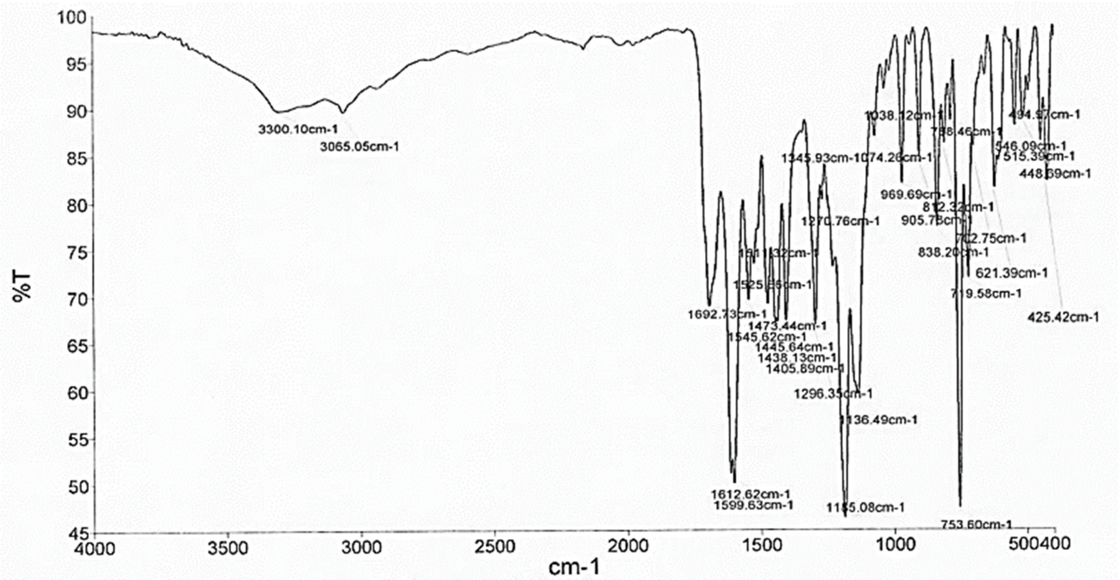

Figure S6. FTIR spectrum of compound **FeL**.

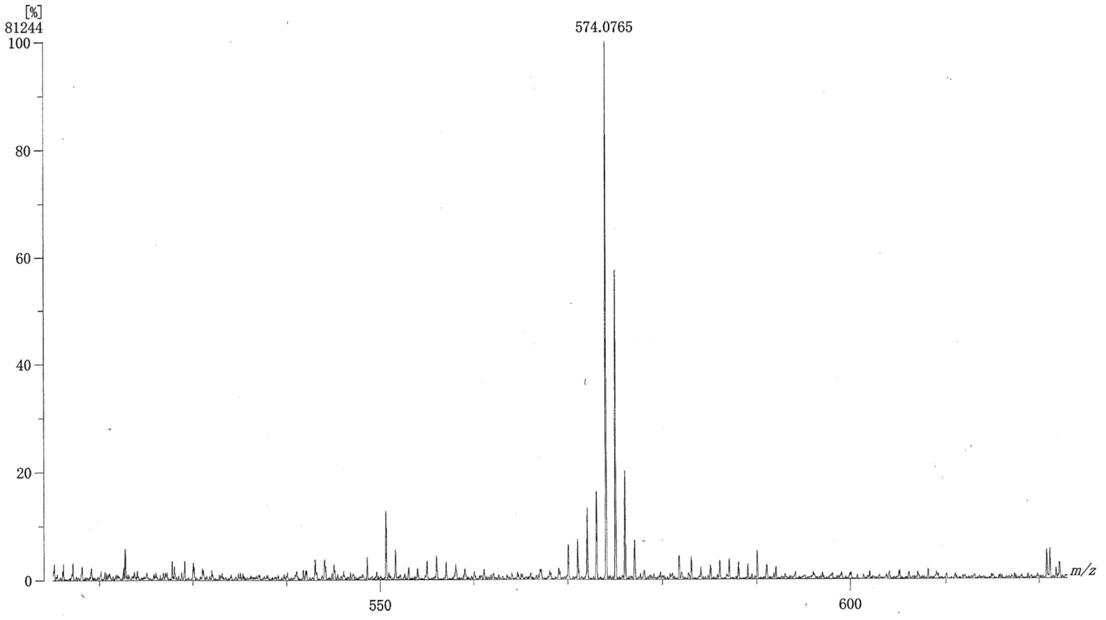

Figure S7. High-resolution FAB mass spectrum of compound **FeL**. Mass spectrum of main peak display parent ion (m/z): calcd, 521.1647 $[M+H]^+$; found, 574.0765 $[M+H]^+$.

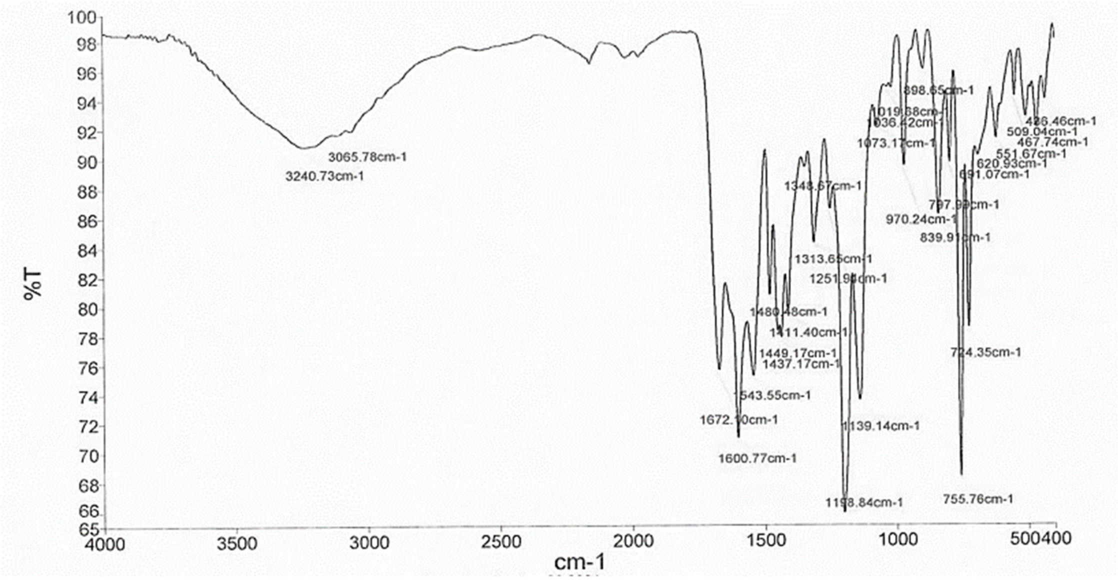

Figure S8. FTIR spectrum of compound **CoL**.

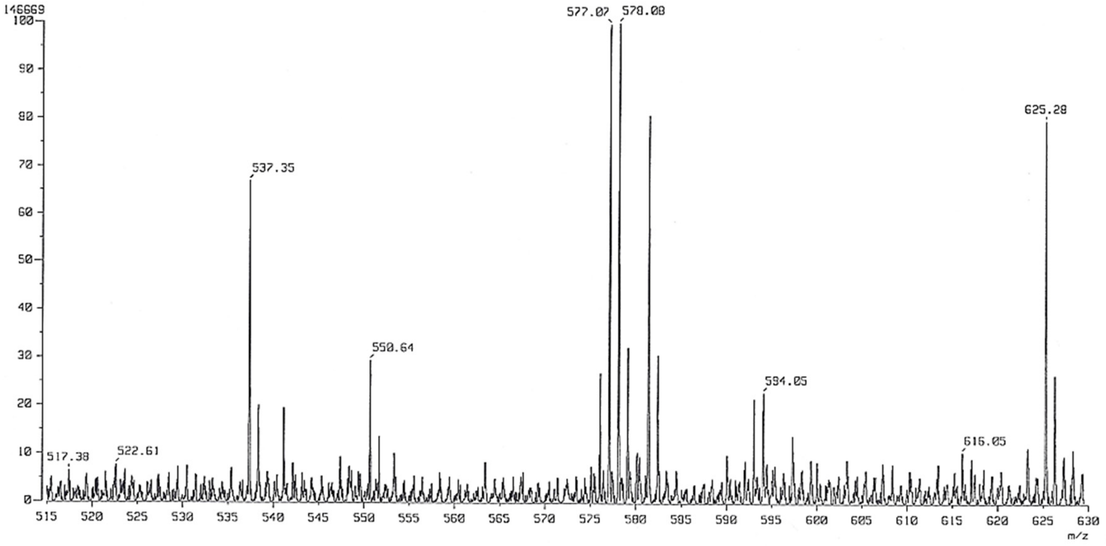

Figure S9. High-resolution FAB mass spectrum of compound **CoL**. Mass spectrum of main peak display parent ion (m/z): calcd, 577.0745 $[M]^+$ and 578.0823 $[M+H]^+$; found, 577.0749 $[M]^+$ and 578.0820 $[M+H]^+$.

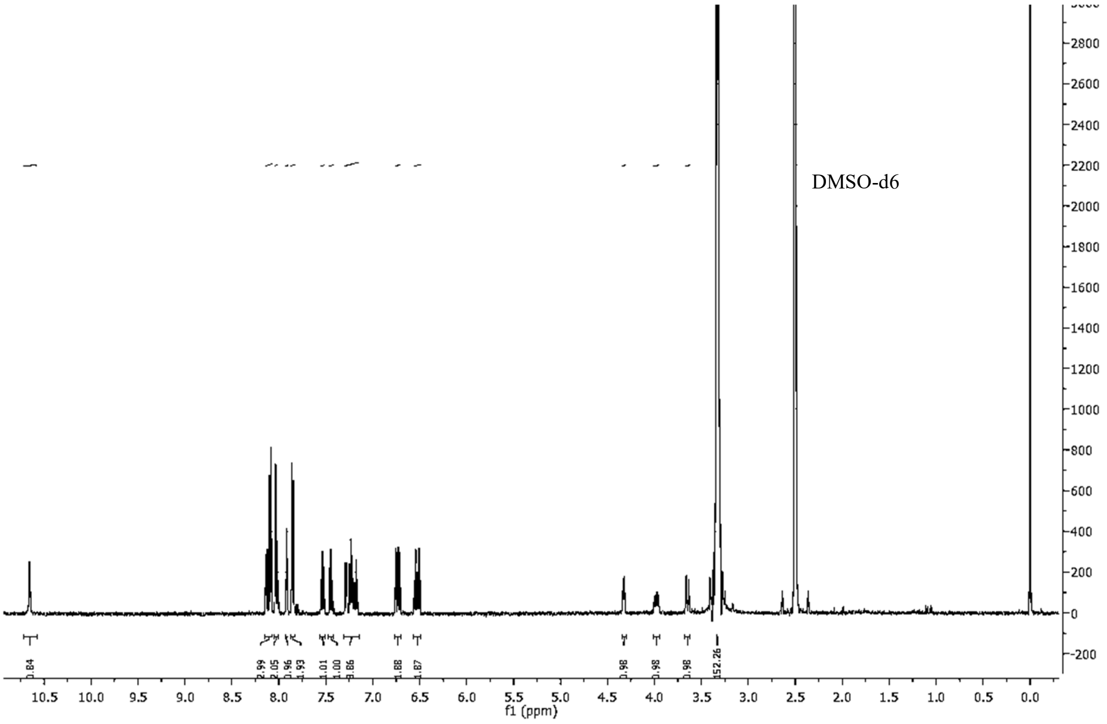

Figure S10. ^1H NMR spectrum of compound **NiL**.

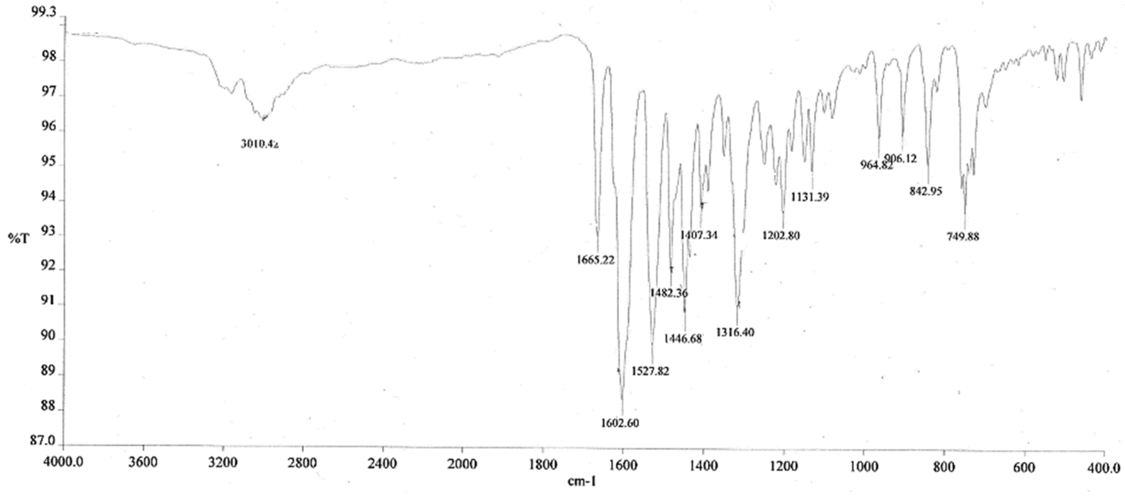

Figure S11. FTIR spectrum of compound **NiL**.

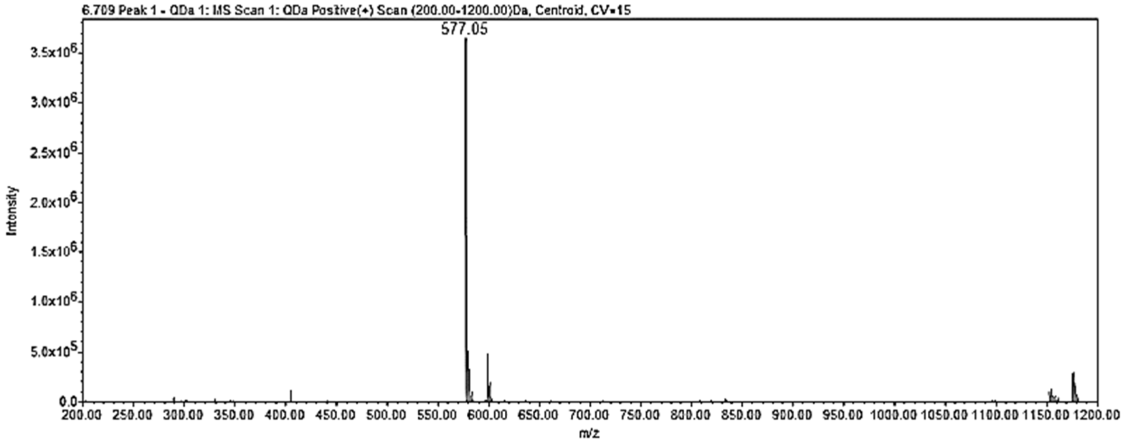

Figure S12. ESI-MS spectrum of compound **NiL**. Mass spectrum of main peak display parent ion (m/z): calcd, 577.08 $[M+H]^+$; found, 577.05 $[M+H]^+$.

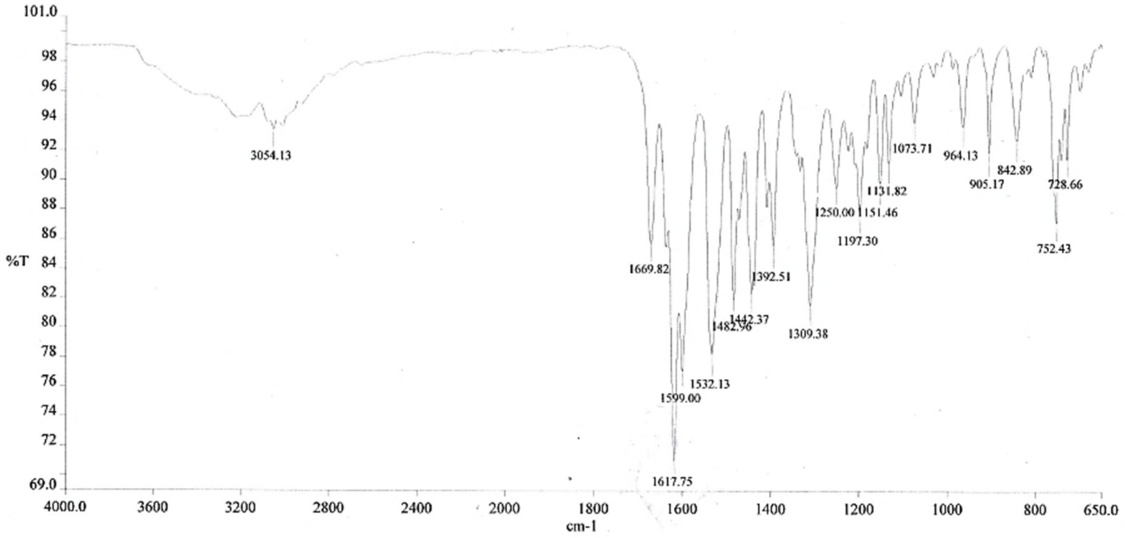

Figure S13. FTIR spectrum of compound **CuL**.

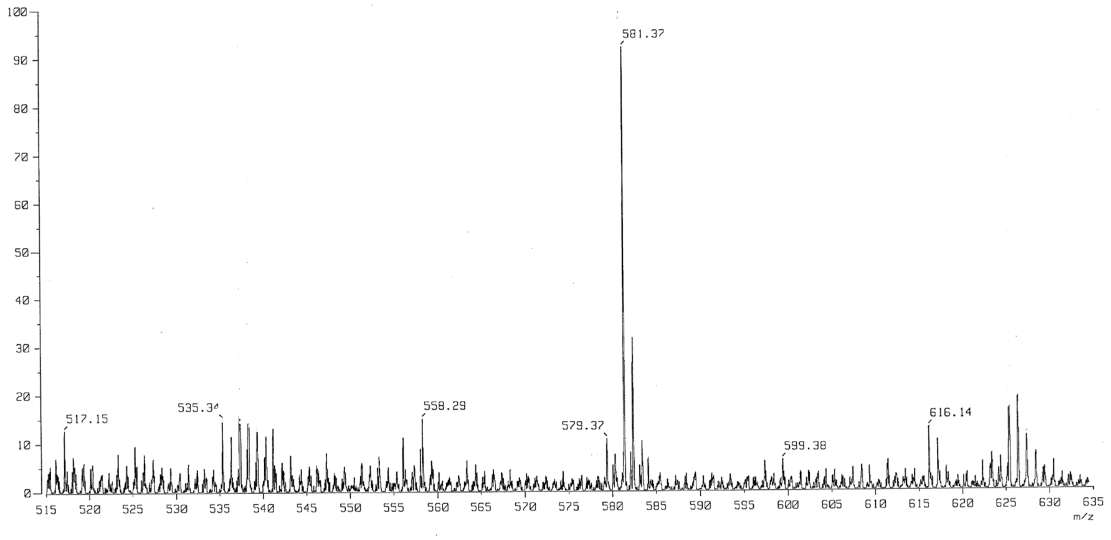

Figure S14. High-resolution FAB mass spectrum of compound **CuL**. Mass spectrum of main peak display parent ion (m/z): calcd, 581.0809 $[M]^+$; found, 581.0709 $[M]^+$.

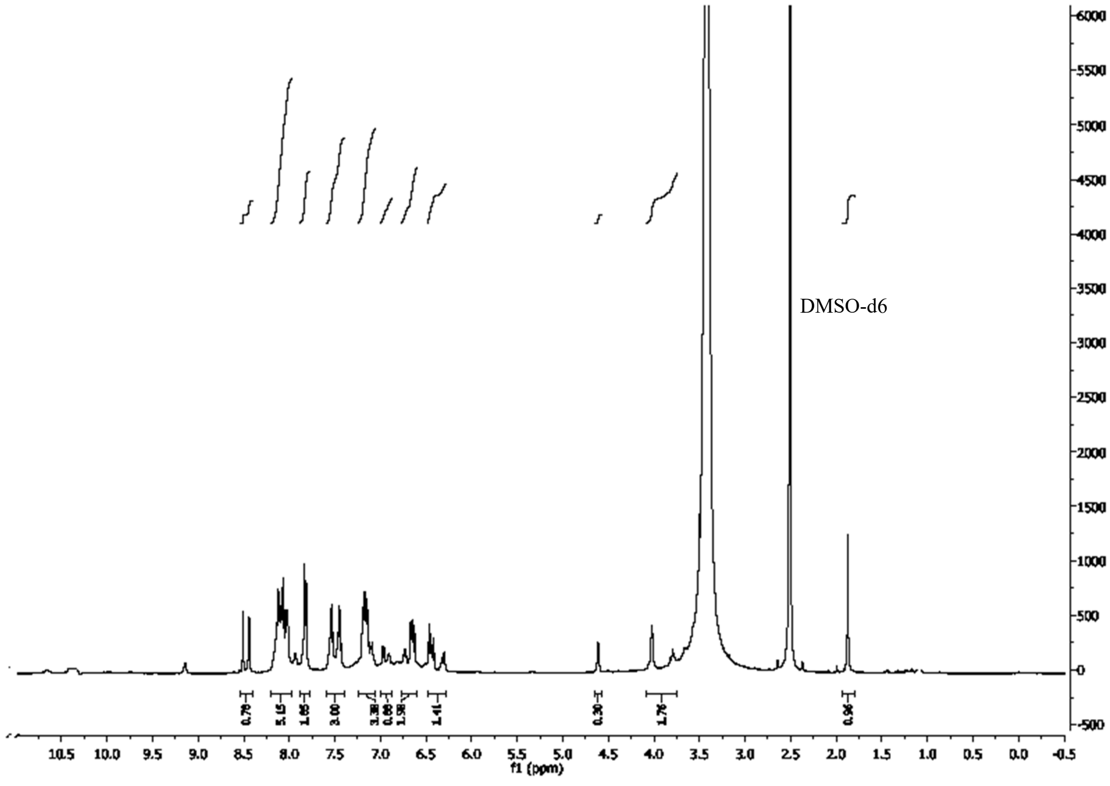

Figure S15. ^{1}H NMR spectrum of compound **ZnL**.

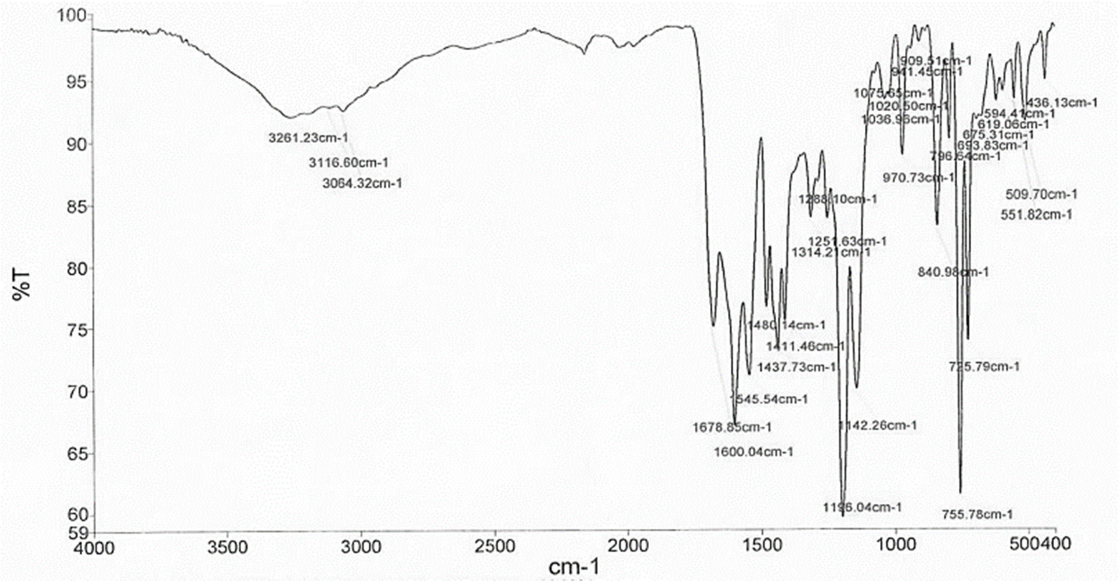

Figure S16. FTIR spectrum of compound **ZnL**.

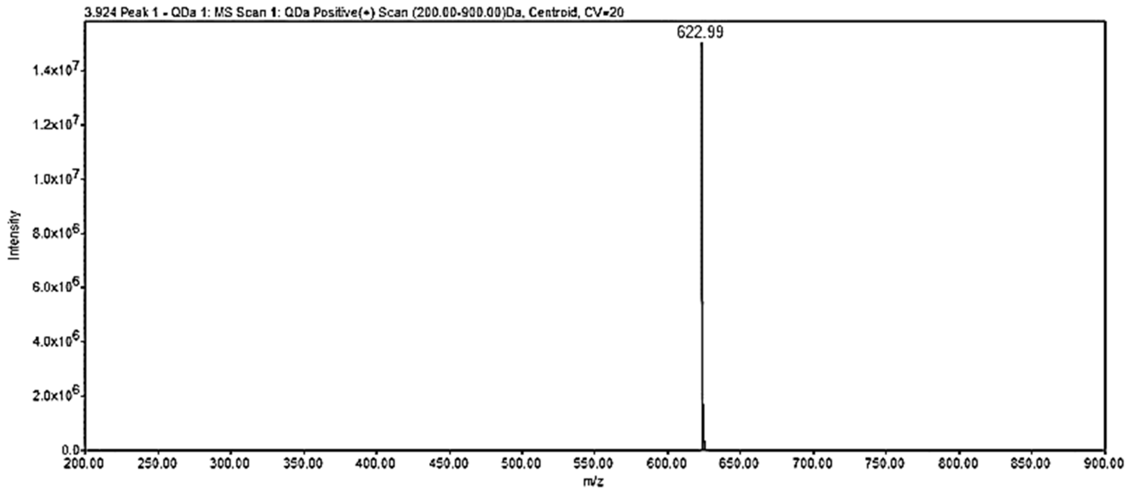

Figure S17. ESI-MS spectrum of compound **ZnL**. Mass spectrum of main peak display parent ion (m/z): calcd, 622.04 [M+K]$^{+}$; found, 622.99 [M+K]$^{+}$.

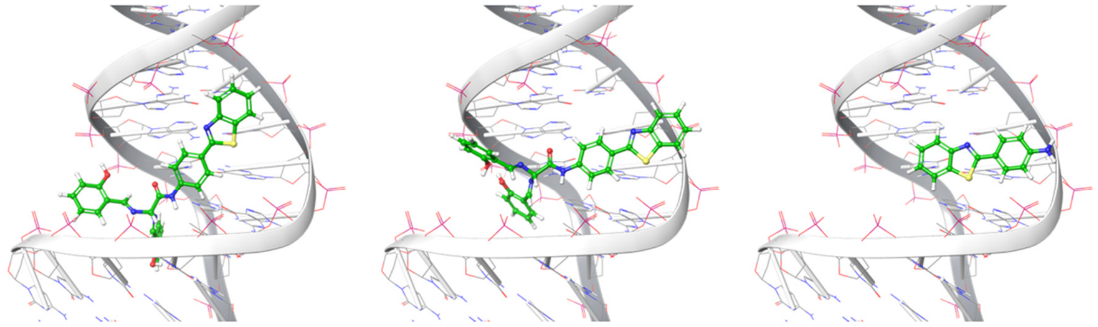

Figure S18. DNA binding configurations were predicted for the compounds (a) **L**, (b) **MnL**, and (c) BTA (PDB ID: 1LU5).

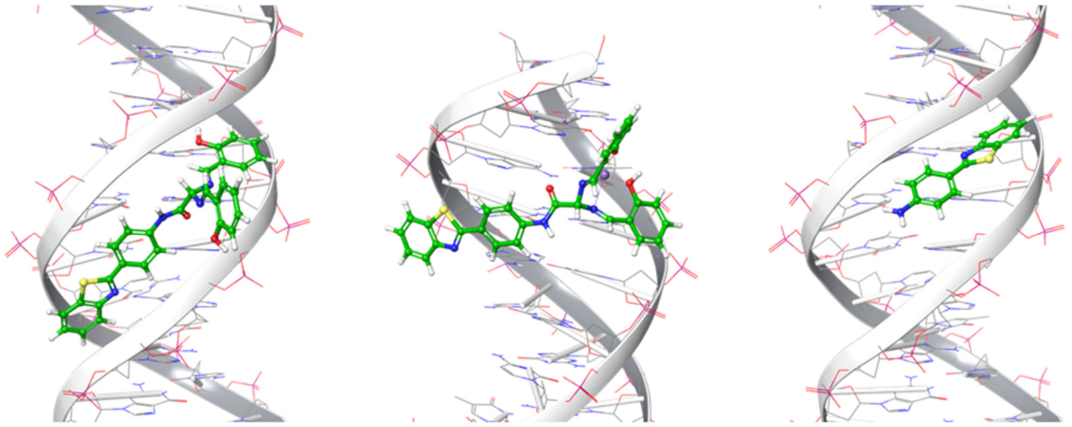

(a) 3CO3-L **(b) 3CO3-MnL** **(c) 3CO3-BTA**

Figure S19. DNA binding configurations were predicted for the compounds (a) **L**, (b) **MnL**, and (c) BTA (PDB ID: 3CO3).
